# Has flood damage being reduced? A resident perspective on the effectiveness of flood management

**DOI:** 10.1371/journal.pone.0325286

**Published:** 2025-06-12

**Authors:** Lumin Hong, Guangwei Huang

**Affiliations:** 1 School of Sociology and Humanities, Jiangxi University of Finance and Economics, China; 2 Graduate School of Global Environmental Studies, Sophia University, Japan; Amity University Amity Institute of Biotechnology, INDIA

## Abstract

Flood disasters have been studied intensively and extensively. However, studies to evaluate the long-term effectiveness of flood management from social perspectives are limited. Questions such as whether flood damage has been reduced or exacerbated have been insufficiently examined and poorly answered from the residents’ view angle. Usually, annual flood damage is used to quantify the economic impact of flood. However, as the estimation of flood-caused damage is affected by various uncertainties in methodology, the development of an indicator without ambiguity is needed for the assessment of flood management effectiveness. Moreover, annual flood damage is often the focus of researchers and administrators. The aim of this paper was to address the often-neglected question of what is the right perspective to better understand long-term changes in flood damage over time, and a related question of what perspective is most tangible to residents and can be used to promote public participation in flood risk management. Thus, the present work used Japan’s flood damage data over the past several decades to analyze various flood damage indices and identify the ones that can be used to detect significant changes in flood impacts without uncertainty, which can also bridge science with residents. The main finding is that the number of flooded houses and semi-damaged houses divided by the annual inundated residential area fit for the purpose of the present study. Since house inundation is more tangible to residents than annual flood economic loss, the findings suggested that the resident-oriented indicator can better reflect the change in flood impact and promote residents’ involvement in the building of coping capacity. In addition, the drivers for flood impact reduction in Japan were discussed and a framework for promoting citizen science for flood risk management was proposed. The overall value of the present work is that it has identified and attempted to fill the knowledge gap in flood impact assessment.

## Introduction

Various natural disasters occur on this planet, of which floods have become the most common [1]. It affects both society and ecosystems in a variety of profound ways, and flood risk is increasing due to climate and socio-economic changes, particularly in Asia and Africa [2]. In China, floods killed more than 282,737 people and damaged 6 billion hectares of land between 1950 and 2018, causing direct losses of approximately $6000 billion between 1990 and 2018 [3]. In Europe, annual flood losses are expected to increase fivefold by 2050 and up to 17-fold by 2080 [4]. Globally, extreme weather events in 2021, including a deep winter freeze, floods, severe thunderstorms, heat waves and a major hurricane, resulted in economic losses of $105 billion, the fourth highest since 1970, according to preliminary sigma estimates by the Swiss Re Institute [5]. Due to the enormous impact of floods and the paradigm shift in flood policy from control to management, the importance of flood damage assessment has become more recognized, as it can support urban planning, building design, climate change adaptation and other future endeavors [6–8].

Flood damage can be divided into direct and indirect damage. Direct damage occurs at the time of flooding due to the physical contact of exposed elements with flood waters, while indirect flood damage refers to the induced losses as a result of flooding, e.g., production losses [9]. The two types of damage can be further subdivided into tangible and intangible. The present study limited its attention to direct flood damage.

Up to now, flood damage assessment approaches may be classified into three categories. The first is to look into depth-damage relationships to quantify flood-induced damage and discuss mitigation measures. The methodologies to develop depth-damage functions include engineering model simulations, curve-fitting on empirical damage or loss data at the individual typology level [10–14], or at the regional level [15]. However, depth-damage functions may have a large uncertainty since actual flood damage data that can be used to calibrate depth-damage models are scarce [16–19], and more fundamentally, flood damage cannot be fully explained by the depth-damage relationship because there are other factors affecting the degree of damage, such as flood water velocity. As a result, applying a depth-damage function of certain original typology to even a slightly different target typology could lead to incorrect damage estimations due to disagreements between typologies and flood situations, resulting in low transferability of depth-damage functions [20]. Therefore, developing adequate depth-damage functions to quantify damage is a constant challenge that stands in the way of flood-induced economic loss estimation. The second category is to combine an ensemble of climate model outputs and river flow models with socio-economic impact models for future global projection [21]. Under a range of temperature and socio-economic scenarios, flood impacts are shown to have an uneven regional distribution, with the greatest losses observed on the Asian continent [22]. The first and second categories are model-based, allowing researchers to better understand the mechanisms of flood-caused economic loss and discuss mitigation measures, and project future losses. The third category is data-based and data-driven, such as constructing a flood impact database [23–25], or using statistical analysis to discern changes at various scales [26–27]. Using long-term data from Japan, a study by Huang [28] indicated that the long-term variations of flood fatalities in Japan and China can be explained by the Kuznets Curve theory, but the curve is not applicable to the annual flood-induced economic loss. Such a data-driven analysis provides policymakers with new insights into the effectiveness of flood risk reduction measures.

Therefore, it can be said that the first and second categories represent the engineer’s point of view towards damage, while the third category mainly provides policy makers with the information they need. What has been missing is the residents’ view toward flood impact. Flood damage makes headlines frequently. However, news coverage is of the aftermath of each disaster, with no long-term perspective on the evolution of damage. The use of existing flood damage data, analyzed in depth, should be pursued to help inform residents of changes and help them and policy makers to take better joint action. It has been reported that citizen science can support flood hazard assessment by providing valuable local rain data [29] and the information sharing via website or social media [30] can fill spatial and temporal hydrological network data gaps. An example is that De Andrade et al. [31] collected Twitter messages combined with official rainfall data to detect rainfall patterns in real time. Nevertheless, the contribution of residents to flood risk management can go beyond basic data acquisition. Fouladi Semnan, et al. [32–33] studied motivational factors for flood-prone residents to adopt resilient-type flood mitigation measures. The involvement of the public in assessing long-term changes in flood impact and mitigation measure implementation is a new arena to be developed, and the present study is aimed at contributing to the development of this arena.

Further, flood damage analysis is often performed at a global, continental, or aggregated multi-country level. Single-country analysis is limited and often focused on the estimation or prediction of annual country-wide total economic loss. Although the annual flood damage at national scale is key information for the evaluation of the effectiveness of government investment on water infrastructure, its suitability for public participation in flood risk management has never been discussed according to author’s literature survey. Information such as the actual inundation area and flood damage in different sectors have received much less attention.

Given that flood economic damage is the social impact of flood disasters, reflecting the success or failure of flood risk management, a research question is how long-term flood damage records could be better used to provide residents with easy-to-understand information regarding the effectiveness of flood management in a region or country and to promote the public involvement in flood impact mitigation. It was hypothesized that different damage indices differ in their ability to show significant change.

Following this line of thinking, the objective of the present study was to explore an effective time series analysis of flood damage data to validate this hypothesis and come up with a recommendation regarding index selection from resident’s view angle. It is also intended to furnish a holistic view about how flood damage in Japan has evolved with time, which can be easily understood by the public. Since the change in flood damage can be viewed as a proxy indicator for change in social vulnerability, consideration of residents’ view will help improve the public participation in flood vulnerability reduction. Finally, it was designed to explore the potential of using flood damage assessment to promote public participation in flood risk management.

## Materials and methods

The present study targets the entire Japan. The Portal Site of Official Statistics of Japan, e-Stat [34], delivers the one-stop service for official statistics of the Japanese government. It is pursuant to “the Optimization Plan of Business Processes and Systems of Statistical Surveys and related work”, which is promoted by the Statistics Bureau, Ministry of Internal Affairs and Communications with collaboration of Ministries and Agencies. Under the Optimization Plan, the Site aims to gather information from statistical departments of Ministries and Agencies, and provides general public with statistical data, schedule of release, etc. The annual flood economic damage, annual inundated area, annual inundated residential area, annual numbers of inundated, completely destroyed and semi-damaged houses were obtained from this site. A semi-damaged house in the disaster management of Japan is defined as a dwelling house that lost part of its basic functions for habitation but can be re-used as it was when repaired. Specifically, where the damaged area is between 20% and 70% of the total floor area of the dwelling house, or where the economic damage to the main components of the dwelling house, expressed as a percentage of the dwelling house’s total value, is between 20% and 50%.

Other data and information such as rainwater harvest facilities and latest policies towards flood risk reduction were obtained from the repository of the Ministry of Land, Infrastructure, Transport and Tourism of Japan and related municipality sites. In this context, preliminary surveys were also carried out to investigate the level of knowledge about rain gardens and the Household Flood Prevention Program among the younger generation, and how to better encourage public participation.

Using data spanning three decades from the 1990s to the present day, changes in flood damage were assessed using Change Point Analysis.

Change Point Analysis has been an active research area since its inauguration in the early 1950s. It is an effective and reliable statistical tool for detecting mean shifts in a time series [35]. It has been largely used in medical science [36]. However, its applications to natural disaster studies were limited. In the present study, this method was applied to both original nation-wide flood damage data and several ratios, such as the annual damage per unit inundation area, the annual damage per unit inundated residential area, etc.

The statistical inference about change points has two aspects. The first is to detect if there is any change in the sequence of observed random variables. The second is to estimate the number of changes and their corresponding locations. The problem of detecting change points is formulated as below

There are many ways to perform change point analysis such as Pettit’s non-parametric ranking test [37–38] and the Buishand’s parametric test [39]. Inclan and Tiao [40] used the Cumulative Sum (CUSUM) method to test and estimate the multiple change points problem, which is a simple but reliable algorithm. For more works related to the change point analysis, the reader is referred to Gupta and Chen [41], Hariz and Wylie [42], Mao et al. [43], and Zhou et al. [44].

The present study employed the CUSUM method, combined with a bootstrapping analysis to detect shifts in the mean of a time-series and confidence levels as developed by Tayor [45].

Let X_1_, X_2_,..., X_n_ be a sequence of independent random variables and X be the mean,


$$X=\frac{X_{1}+X_{2}+\ldots +X_{n}}{n}$$ (1)

Set S_0_ = 0

Si are computed recursively as follows


$$S_{i}=S_{i-1}+(X_{i}-X)$$ (2)

The cumulative sums are not the cumulative sums of the values, but the cumulative sums of differences between the values and the average. These differences sum to zero so the cumulative sum always ends at zero.

The confidence level is determined by performing a bootstrap analysis [45–46]. Before performing the bootstrap analysis, the magnitude of the change is estimated below, which works well regardless of the distribution and despite multiple changes according to Tayor [45]


$$△=MaxS_{i}-MinS_{i}(1\leq i\leq n)$$ (3)

After the estimation of the magnitude of the change, the bootstrap analysis is performed, which generates a bootstrap sample of n data points of time series by randomly reordering the original values. This is called sampling without replacement. Then, based on the bootstrap sample, the bootstrap CUSUM is calculated following same method. The difference between the maximum and minimum bootstrap CUSUM is defined as


$${△}_{B}=MaxS_{j}-MinS_{j}(1\leq j\leq n)$$ (4)

The next step is to check if the bootstrap difference is less than the original difference.


$${△}_{B}<△$$ (5)

The bootstrap analysis consists of performing a large number of bootstraps and counting the number of bootstraps for which bootstrap difference is less than the original difference. Let N be the number of bootstrap samples performed and K be the number of bootstraps for which

${△}_{B}<△$. Then the confidence level that a change occurred as a percentage is calculated as follows


ConfidenceLevel=100(K/N) (6)

**Fig 1:**
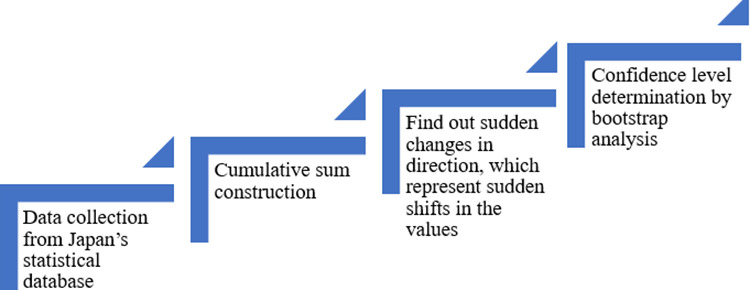
Flowchart of the change point analysis.

Once a change point has been detected, an estimate of when the change point occurred can be made by locating the point furthest from zero in the CUSUM chart, which represents the last point before the change occurred. the mean square error can also be used as a second estimator of when the change occurred. The above-described change-point analysis procedure is reasonably robust to statistical outliers. If data contains outliers that will cause change points to be missed, the analysis can be performed to eliminate the effects of outliers by analyzing the ranks of the values instead of the values themselves. The flowchart of the method was presented in Fig 1. The analysis was performed using Change-Point Analyzer [47], which is executed using C^++^. It is worth mentioning that the change point analysis in R is available online as described by Killick and Eckley [48].

By applying the change point analysis to different data. the right indices for evaluating the effectiveness of flood risk management were proposed, and major challenges Japan must take to further reduce flood risk were highlighted.

In addition, preliminary social surveys were conducted and the results were used to identify ways of improving public participation, particularly among the younger generation.

The overall value of the present study is that it provided a new diagnosis of how flood damage has changed with time and found the right index for trend detection, which is tangible to residents so that it can be used to facilitate public participation in flood risk management.

## Results

**Fig 2:**
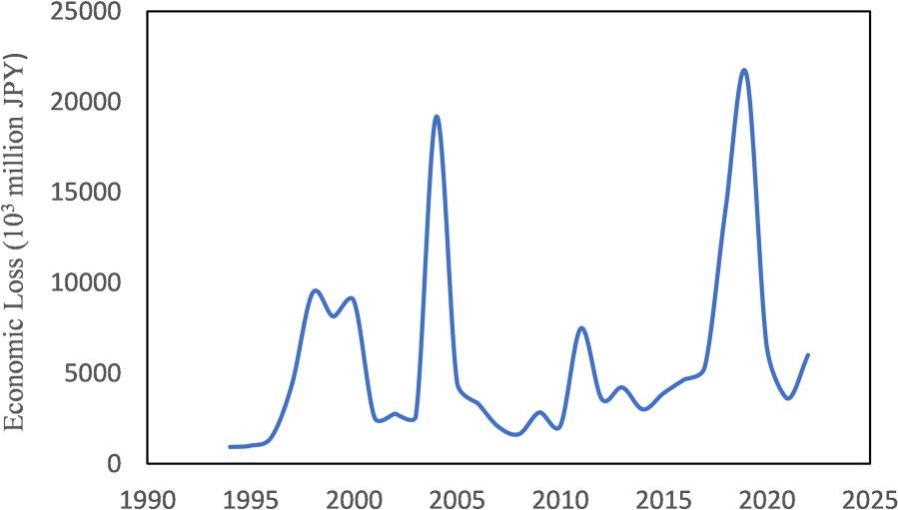
Annual flood damage in Japan.

Fig 2 shows the change of total annual flood damage in Japan over the past three decades. It has a wave-like variation with two highest peaks in 2004 and 2019, respectively. In 2004, ten typhoons landed on Japan, which is the largest number on record. In the same year, Japan was also hit by two separate torrential rains in July. These rainfall events resulted in the collapse of riverbanks along the Ikarashi, Kariyata and other rivers in the Shinano River system and the Asuwa and other rivers in the Kuzuryu River system [49]. In 2019, Typhoon Hagibis made landfall in the Izu Peninsula, and the storm cut through the eastern part of the island of Honshu, causing 140 levee breaches along more than 70 rivers throughout the country [50]. Although the annual flood damage appears rising, the change-point analysis detected no point of significant change.

**Fig 3:**
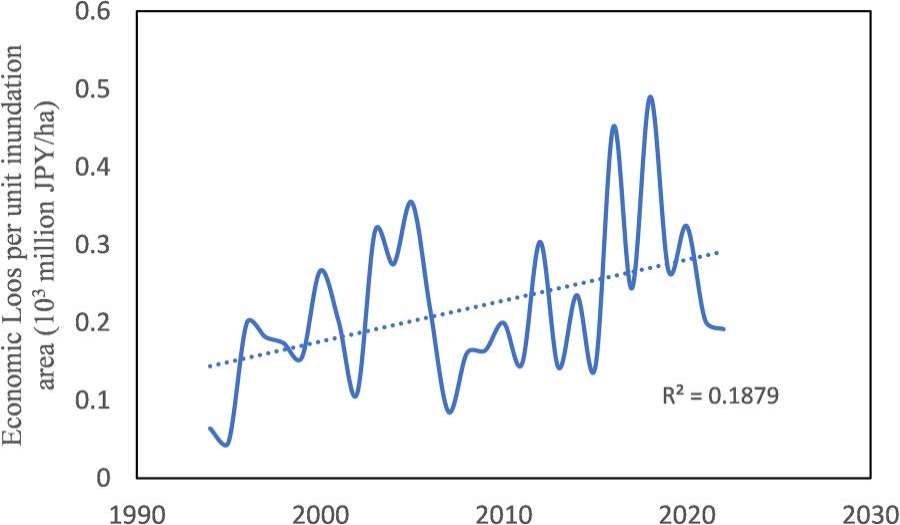
Annual flood damage normalized by annual nationwide inundated area.

**Fig 4:**
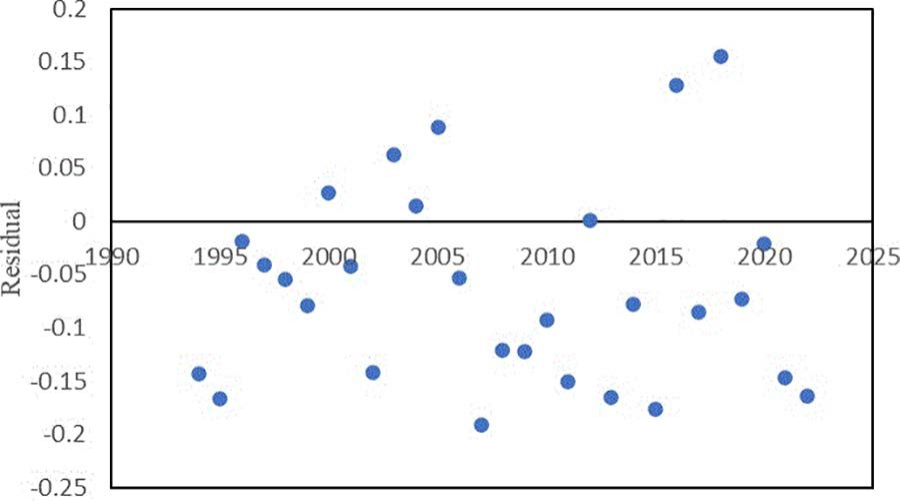
Residual plot.

Fig 3 shows the annual change in the ratio of annual damage to the annual nationwide inundated area. When looking at this figure, it is tempting to think that there is an upward trend. Despite this, the linear trend analysis revealed a very low R^2^ value. Besides, the residual plot (Fig 4) shows that although residuals are close to zero, they either increased or decreased with time, without showing a random distribution. Moreover, the change-point analysis did not detect any significant change point.

**Fig 5:**
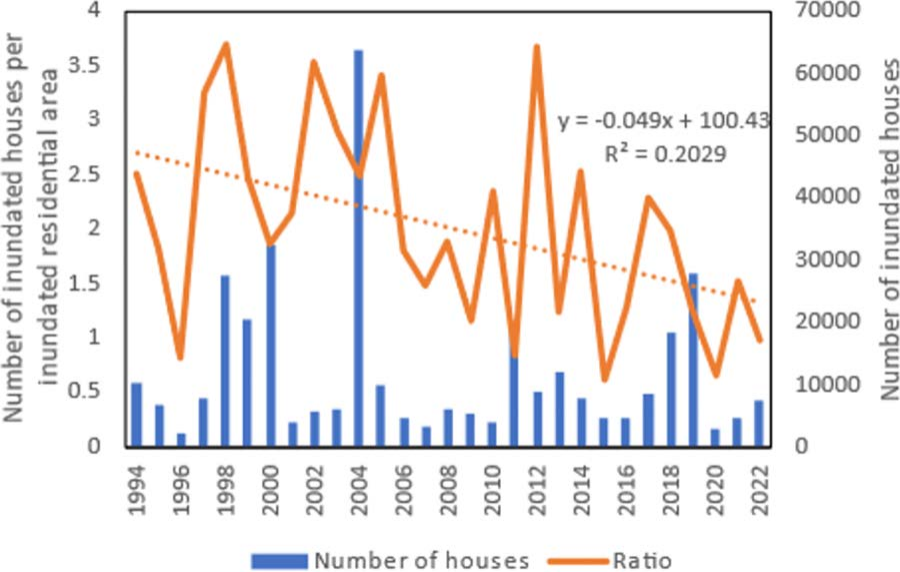
Variations of the annual number of houses inundated with water depth deeper than their floor height and the ratio of the number to the annual inundated residential area.

Fig 5 shows the variations of the annual number of houses inundated with water depth deeper than their floor height, which is 45 cm according to the Japanese Building Standards Act. It also shows the ratio of the number of inundated houses to the annual inundated residential area. For the number of inundated houses, no trends were detected. For the ratio, although a linear trend analysis indicated a low R^2^ value as shown in Fig 5, the change-point analysis detected a point of significant change in 2006 as compiled in Table 1. When the number of houses with water depth deeper than their floor height is divided by the total flooded area nationwide, no significant change point was detected. These results confirm the hypothesis that different damage indices differ in their ability to detect significant changes. It can also be observed from Fig 5 that the change point for the ratio occurred two years after the peak of the number of houses inundated. This finding shed new light on the effectiveness of flood impact mitigation measures and merits further attention.

**Table 1:** Significant change point in the number of flooded houses per unit inundated residential area.

Year	Confidence interval	Confidence level	Shift From (Mean number of occurrence)	To
2006	(2006, 2012)	96%	2.602	1.617

Next, the change point analysis was applied to the ratio of the number of completely destroyed houses to the annual inundated residential area. However, no change point was detected. A further step was the application of change point analysis to the number of semi-damaged houses and the ratio of the number of semi-damaged houses to the annual inundated residential area. For the number of semi-damaged houses, no change point was detected. For the ratio, a point of significant change was detected in 2004, as compiled in Table 2.

**Table 2:** Significant change point in the number of semi-damaged houses per unit inundated residential area.

Year	Confidence interval	Confidence level	Shift From (Mean number of occurrence)	To
2004	(2004, 2-11)	97%	0.062	0.589

**Fig 6:**
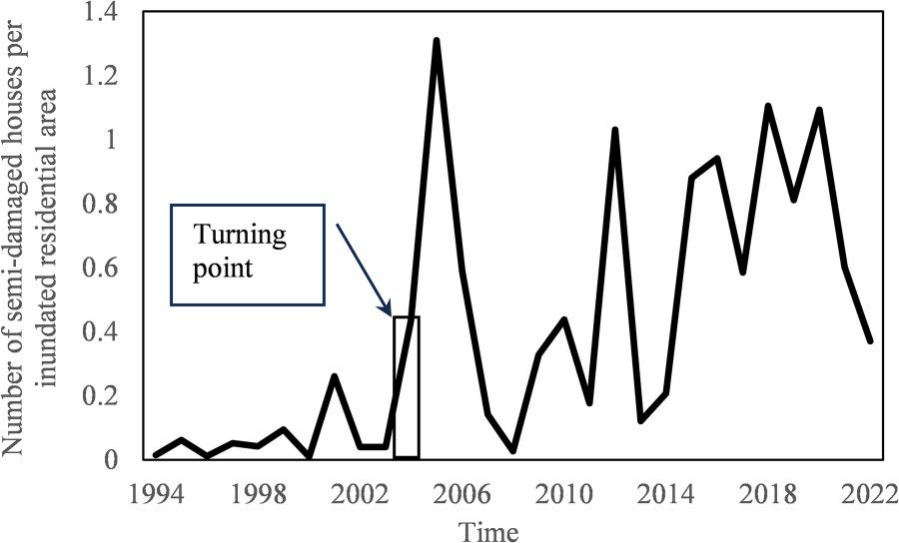
Variations of the ratio of the number of semi-damaged houses to the annual flooded residential area.

As shown in Fig 6, the ratio of the number of semi-damaged houses to the annual flooded residential area shows an upward trend, and the turning point of the upward trend occurred before the turning point of the downward trend for the ratio of the number of flooded houses to the annual flooded residential area. This finding indicates that flood risk management measures have led to an overall reduction in the number of houses flooded, but have not been able to reverse the trend of severe damage to houses. This situation can be described as a bipolarization of flood impacts. In other words, while there has been a reduction in the number of houses flooded, the number of houses with severe damage has been higher.

The number of houses inundated and heavily damaged is the top concern for residents although the damage to infrastructure, agriculture and business facilities can be considered more critical to a nation’s economy. The findings of this work suggested that although Japan’s economic vulnerability to flooding remains statistically unchanged, the overall residential property damage has been significantly reduced when normalized properly. However, this success was cancelled by the fact that the number of severely damaged houses has been rising. This may partially explain why economic vulnerability to flooding remains statistically unchanged. Apart from the death of a loved one, damage to the home is a major concern for individuals. Therefore, in parallel to the national economic loss, what is most important to the residents should be given the same priority in the management of the flood risk. The results of this work suggested that it is necessary to use a correct denominator for the index calculation in order to better inform residents about changes in property damage over time.

## Discussions

The downward shift in the ratio of the number of inundated houses to the annual inundated residential area is a result of various flood damage reduction measures such as house raising work, watertight board installation, the installation of rainwater storage tanks and so on. In Japan, rainwater harvesting facilities have been promoted since the 1980s. When rainwater is stored in houses and buildings, it can be used for daily watering, car washing, etc., as well as for daily use in emergencies such as disasters, and it also helps prevent urban flooding by reducing the runoff of rainwater. Furthermore, various stormwater retention facilities have also been constructed in various places. In Tokyo, there is a massive underground regulating reservoir built under a major ring road that runs around central Tokyo. It can store approximately 540,000m^3^ of flood water from the Kanda River and the Zenpukuji River. It functions as a critical buffer, protecting both residential and commercial areas from the devastating impacts of floods. There are also many small rainfall retention facilities such as rain gardens. In 2006, Saitama Prefecture enacted an ordinance for the installation of rainwater runoff control facilities [51]. It stipulates that in the case of development activities of 1 hectare or more, it is necessary to install a rainwater runoff control facility with permission from the governor.

**Fig 7:**
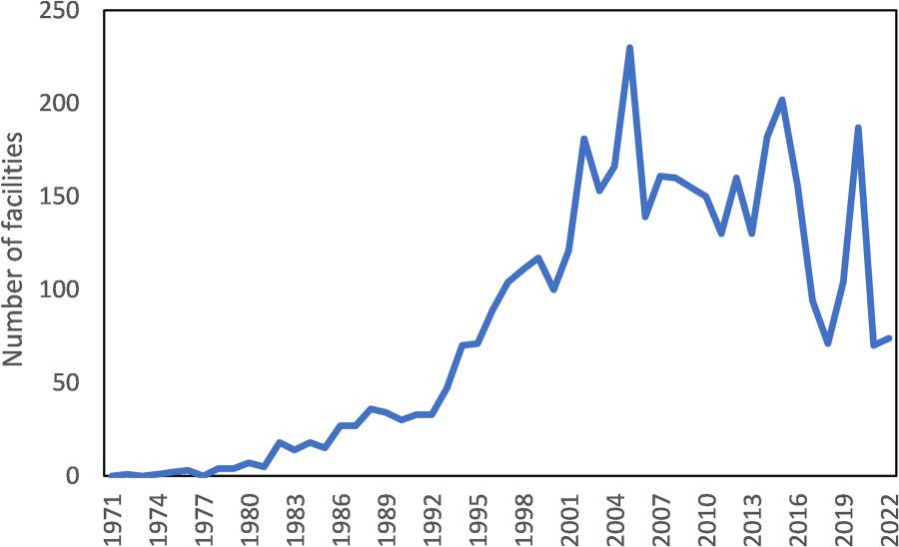
The number of rainwater harvesting facilities installed per year from 1971 to 2022.

Such a regulation has now been adopted for the management of important watersheds. Fig 7 shows the number of rainwater harvesting facilities installed per year from 1971 to 2022. The number peaked in 2005. Three years later, a turning point of over-floor flooding was reached, which may be partially attributed to the effect of rainwater harvesting and retention facility installation. Although there have been reviews of rainwater harvesting systems in the literature [52–53], the present study shed new light on their effectiveness on a national scale.

In the 1990s, the Japanese government initiated a program under the policy of resilient river management, which was named Special Emergency Program to Prevent House Inundation [54–55]. The program specifies that in areas where flooding of residential houses occurs frequently, flood control approaches should be integrated and consolidated to eliminate the chronic flooding in five years. The eligibility for a project funding application under the program is given below

- More than 200 houses have been flooded with flood water depths less than 0.5 m in the past 10 years
- More than 50 houses have been flooded with flood water depths more than 0.5 m, or subways, underground malls, power plants, and substations stopped functioning due to flood damage in the past 10 years.

In 1995, the construction of the Nakae River flood regulating pond was adopted as a project under the special emergency program. With the completion of this project in 2004, the potential inundation area in the Nakae River basin was estimated to be reduced from 137 ha to 43 ha [56].

A recent case is that after a flood disaster occurred in the Tsubo River basin in 2018, a special emergency project was carried out along the Tsubo River. In 2023, the river basin was hit again by heavy rainfall of similar intensity, but the number of houses inundated was reduced from 360 in 2018–0 in 2023 [57].

Both rainwater harvest and house inundation prevention were designed to reduce surface runoff, and their message is clear to residents.

In the 1990s, a management objective of the Japanese government was set to reduce or even eliminate chronic residential house inundation by 2000. The present study found that initiatives taken in the 1980s and 1990s did bring about significant improvement, although the turning points occurred several years behind schedule. This finding provides the first concrete evidence that the rainwater harvesting initiative, and the house inundation prevention program together have made a difference.

**Fig 8:**
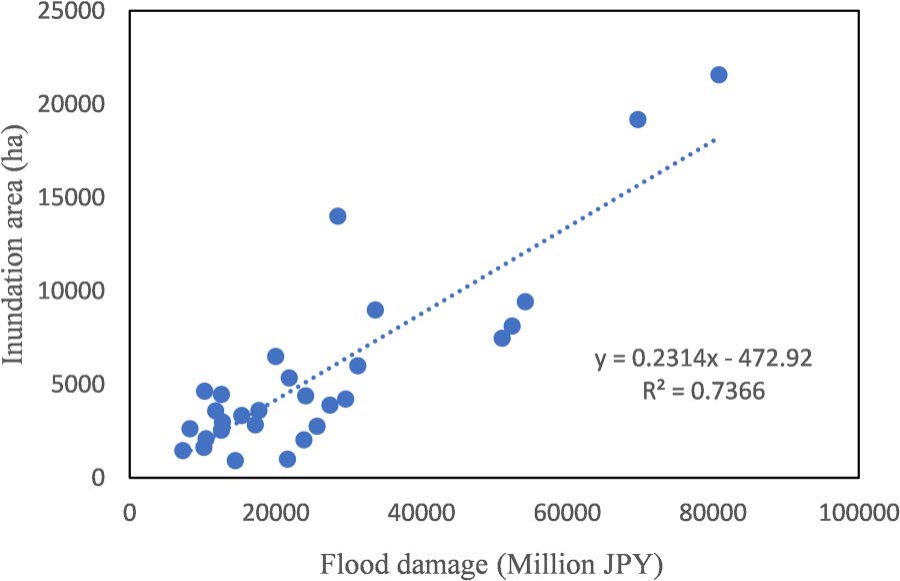
Correlation between annual flood damage and inundated area.

**Fig 9:**
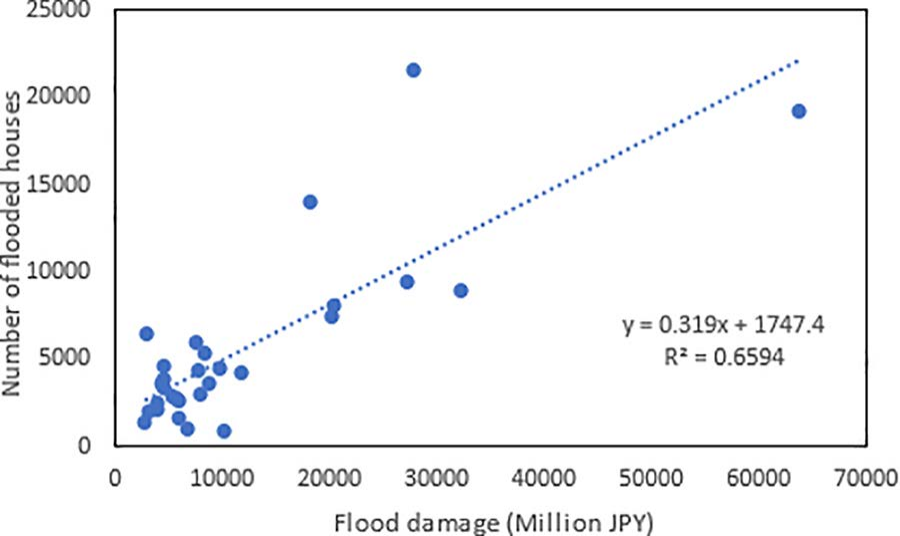
Correlation between annual flood damage and the number of houses flooded.

Up to now, there is a general perception among Japanese that the flood-caused economic damage in Japan has not decreased. The reason is often explained as the concentration of people and assets in flood-prone areas. Life-saving can be achieved through better evacuation, but property damage is difficult to avoid as long as it is located in high risk zones. However, so many countermeasures either structural or non-structural have been implemented in Japan, why these efforts did not lead to a significant reduction in economic loss deserves in-depth analysis. The present study revealed two reasons. One is that the index we have been using to quantify flood damage does not have a right denominator, so that it could not provide a complete picture of how flood damage changes with time. Although the annual damage or the annual damage per unit flood area does not show a decline trend, the number of inundated houses per unit inundated residential area has a downward shift as revealed by change-point analysis, and this shift can’t be found by linear trend analysis. Another reason is that the number of semi-damaged houses per unit inundated residential area has been increasing. The decrease in the number of flooded houses per unit of flooded area was offset by an increase in the number of semi-damaged houses per unit of flooded area. Besides, another perception in Japan is that inundated areas have been decreasing while flood damage intensity has been skyrocketing. However, the change-point analysis performed with the data of the annual inundation area from 1994 to 2022 found that the annual inundated area in Japan did not exhibit a decreasing trend. Indeed, Fig 8 shows that the annual flood damage and inundated areas are positively correlated. This situation is due to the fact that 75% of the total assets of Japan are concentrated in flood-prone areas. The relationship between the number of houses flooded annually and the annual flood damage was also examined. As shown in Fig 9, the residential property damage accounted for 65% of the variation of the annual flood damage. Since the number of houses inundated is a function of water depth and is well correlated with the annual flood damage, it implies that high inundation water depth is a major factor regulating the damage. Therefore, new countermeasures should be explored to further reduce flood water depth in inundated areas, which will lead to more flood damage reduction. Clarification that flooded area has not decreased in Japan contributes to correct flood risk perception and better policy making.

**Fig 10:**
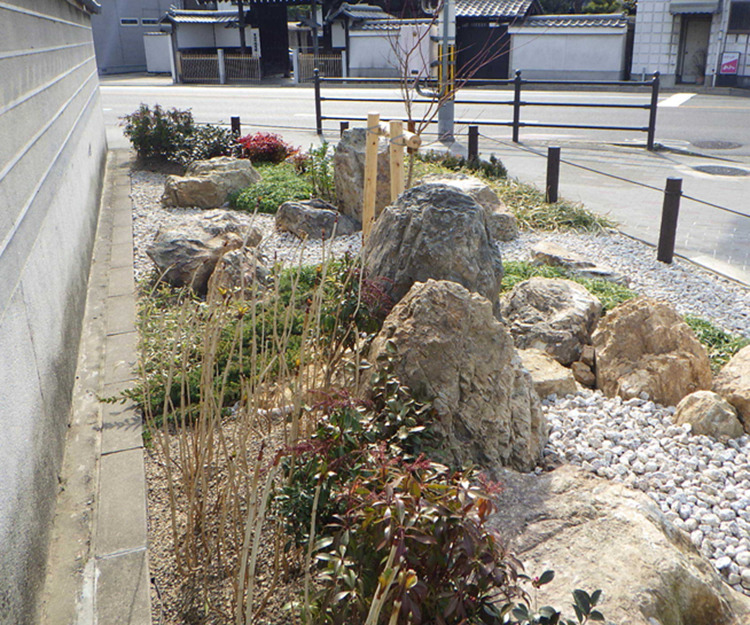
A rain garden in Kyoto.

Both rainwater storage and house inundation prevention program are good triggers for the public participation in flood risk management. In 2022, Setagaya City in Tokyo Metropolitan established a “Guidelines for the Installation of Rainwater Runoff Control Facilities in Setagaya City”, which is a subsidy system to encourage residents to install rainwater tanks [58]. At the same time, Kyoto City is playing a leading role in promoting the “Rain Garden”. Rain gardens, also known as bioretention cells, consist of a depressed area with plants, an engineered soil mixture, and a drainage bed that meets the water reuse requirement. When stormwater enters the rain garden surface, it can be temporarily stored in the hollow space, which may alleviate waterlogging and relieve the pressure on urban drainage systems during heavy rains. On May 10, 2022, a sample rain garden was completed in front of Kyoto City Hall. The rain garden, which combines Kyoto’s traditional landscaping culture and technology with flood countermeasures, is attracting attention as a new form of disseminating the message of green infrastructure. An innovative practice taken by Kyoto City is that it constructs rain gardens along with roadside trees and uses the existing “Kyoto City Street Tree Supporter System”, which is a system to promote volunteers’ activities and recruit volunteers to conduct maintenance work for both rain gardens and trees [59]. From 2022 to 2024, 14 rain gardens were built in Kyoto City. Nevertheless, our survey using Google Maps and street view found that all rain gardens in Kyoto City were constructed in public spaces. A photo of a rain garden in Kyoto taken by the authors of this paper is shown in Fig 10. This suggests that new policies are needed to encourage residents to set up rain gardens in their backyards.

Using a rain garden of 0.5 m deep, Ishimatsu [60]reported that the infiltration capacity per 1m^2^ is 0.07 m^3^/min. It also suggested that rain gardens must be connected to conventional sewage systems and they require maintenance work. The most frequent maintenance is the disposal of garbage, which flows into rain gardens on rainy days. Garbage may retain rainwater for several days, which could become a habitat for mosquito larvae. Besides, the removal of invasive/nonnative plants, pruning of overgrown plants, and gardening for aesthetic value are also required, depending on the vegetation composition of a rain garden. Therefore, design standards for low construction cost, self-watering and self-fertilizing rain gardens should be developed. Universal design can be cost-effective and easy to spread. On the other hand, using storm sewage to reduce urban flooding has been steadily promoted. In areas where populations and assets are concentrated, and severe damage has occurred, the coverage of storm sewage has reached 59% as of the end of 2018. However, the present design standard is the rainfall that occurs once every five years. In light of such a relatively low standard for urban storm drainage, the necessity of rainwater harvest and the house inundation prevention program is justifiable.

In July 2024, a purposive questionnaire survey was conducted at Sophia University. It is a purposive sampling, targeting the younger generation. The main questions to respondents are if they have heard about rain garden and the house inundation prevention program. It distributed 100 samples and collected 53 replies. 95% of respondents are young students. For the rain garden, 10% replied Yes while for the house inundation prevention program, 100% replied never heard about it. This suggested that more surveys should be conducted to clarify the public awareness of flood impact mitigation measures. In early 2025, another survey was conducted at Sophia University and Jiangxi University of Finance and Economics in China, asking young students whether they would find it more appealing to participate in activities for reducing home flooding than activities for reducing national economic losses. It distributed 100 samples in each university. The surveys were carried out in accordance with Ethical Guidelines for Social Research in Japan and informed consent was obtained from all respondents. The responses from Sophia University and Jiangxi University of Finance and Economics were 90 and 88 respectively. At Sophia, 85% said yes, while at Jiangxi, 61% said yes. The reason why the majority chose home flooding is because it is a more personal and safety issue, which is seen as a top priority. This led to the hypothesis that reducing house flooding is a better way to encourage public participation in flood risk management than targeting economic losses at the national level.

Fouladi Semnan et al. [32] examined motivational factors for residents in flood-prone area to adopt resilient type flood mitigation measures such as house elevation and wet flood-proofing. It was found that the flood-coping appraisal and prior flood experience were the primary factors influencing decision-making by flood-prone residents to adopt resilient mitigation measures. By utilizing the cognitive process of Protection Motivation Theory (PMT) and integrating it with the Health Belief Model (HBM), Fouladi Semnan et al. [33] showed that Benefit/barrier Appraisal, Social Environment and the experiences with floods inside buildings are a significant factor influencing the flood-coping appraisal.

In Japan, a devastating disaster occurred in Mabi Town, Okayama Prefecture in 2018. In 2021, a questionnaire survey was conducted by Fujii and Kondo [61] to investigate the recovery. It was found that before the disaster, 68.3% of the respondents had not taken any measures to make their houses flood-proof, but after the disaster, the percentage decreased to 52.8%. Among the flood disaster prevention measures, the shift from one-story to two-story houses is important in terms of vertical evacuation. However, the survey revealed that before the disaster, 5.9% of the houses in the study area were single-story, but after the disaster, the percentage of single-story houses increased to 11.3%. Financial constraint was considered to be the main reason. Another possibility is that it is an elderly town, so it is difficult for elderly people to use the second floor. In other words, the convenience of daily life outweighs the need for disaster prevention. Therefore, the relationship between the experiences with floods and flood coping capacity building can be complicated by income and population structure.

Household income is an important determinant of flood preparedness [33,62,63]. Higher-income households are generally better equipped to anticipate, withstand, and recover from flooding. Addressing this disparity is essential to promoting equitable disaster resilience. Between July 18, 2023, and July 24, 2023, a general information website (All About) conducted a survey on flood disaster awareness among the site’s users [64]. According to the survey, more than 80% of high-income households with an annual income of 10 million yen or more are aware of disaster prevention when buying a house. On the other hand, the percentage is less than 70% for those with annual incomes of 5 million yen or less. It can be postulated that higher-income households are more likely to own homes in areas with better infrastructure and lower flood risk, to retrofit homes with flood-resistant measures (e.g., waterproofing, elevating appliances), and to invest in flood insurance. Japan’s disaster-related insurance system includes “Kasai Hoken” (fire insurance), which can be extended to cover water damage. However, flood-specific insurance under Kasai Hoken is optional and may be unaffordable for low-income households, who often opt for cheaper policies with limited coverage. According to the General Insurance Rating Organization of Japan, 63% of fire insurance subscribers in Japan in 2023 have water damage coverage [65].

Such existing knowledge supports the proposition that public participation in flood risk management can be better encouraged and may lead to better results if the focus is on residential flooding. At the same time, it suggests that it is important to consider more factors in an integrated manner when promoting public participation for resilience building.

**Fig 11:**
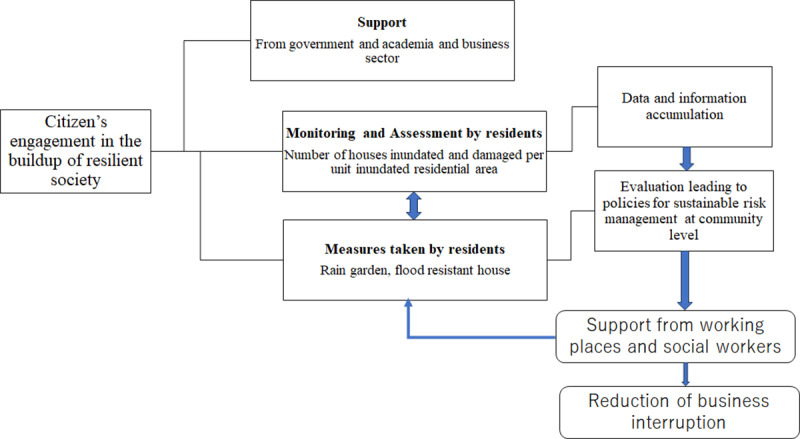
A new framework to promote citizens’ engagement in flood risk management.

Based on the results of the present study and discussions above, a new framework combining monitoring, mitigation initiatives and long-term assessment is proposed to promote citizens’ engagement in flood risk management, as shown in Fig 11. It consists of three components. The first one is the support from government, academia and business sectors. Under the support, the second component is the monitoring and assessment of flood impact by residents using easy-to-use indices such as the ratio of the number of inundated houses to the inundated residential area. Information on residential property damage and inundated residential areas is published by municipalities after each disaster and is readily available to residents. Based on the monitoring and assessment, the third component is the promotion of measures taken by residents, such as turning their backyards into rain gardens or remodeling houses to be flood proof. When a large number of houses install rain gardens or remodeled, residents may use water level loggers to monitor the change in flood water depths on streets and around their houses. As a large amount of data including inundation depth, topography, soil and plant on rain gardens and house conditions are collected and shared, the effectiveness of rain garden and house remodeling in alleviating house flooding can be evaluated as a joint work between residents and professionals.

Financial support from governments, technical support from professionals, and moral and material support from workplaces are essential to involve residents in the process of building a resilient society and to implement residents’ initiatives. This is why it is viewed as the first component of the framework. What is often neglected is that the care of safety and encouragement for disaster preparation given to employees from the working places can play an important role. According to Psychological Contract Theory [66], if a company adopts a people-oriented management mode and takes care of its employees in terms of compensation and benefits, working conditions and environment, job security, promotion opportunities, etc., then the “reciprocity principle” in the exchange of social interpersonal relationships (including social psychology and social behavior) may lead employees to be more inclined to repay the company with higher organizational commitment and work enthusiasm, which in turn generates more organizational citizenship behavior (OCB). Therefore, helping employees financially to prepare for flood risks on normal days, and providing them with supplies and assistance to rebuild their homes after a flood disaster, will benefit the company by making their employees more loyal to the company in the future, more motivated to work harder, and more willing to devote their talents and energy to the company. As a result, the risk of business interruption will be reduced.

The results of resident initiatives can be used to develop new policies to mobilize more corporate support and facilitate social work assistance. Loss of life and property damage can have serious mental and physical health consequences, such as post-traumatic stress disorder (PTSD). Disaster social work can provide unique, life-saving and mental recovery support. New policies are needed to enable social workers to use innovative problem-solving techniques to best support those in need.

## Conclusions

This study examined flood damage in Japan over the past few decades to identify trends, with the aim of better informing the public about changes and promoting public participation in flood risk management. The main findings are summarized below

1. Annual flood damage, annual flood area and annual flood damage per unit inundated area in Japan do not show any significant change with time over the past three decades.
2. By contrast, the number of flooded houses divided by the inundated residential area shows a downward shift that can’t be easily detected by linear trend analysis.
3. However, the number of semi-damaged houses divided by the inundated residential area shows an upward trend.
4. The coexistence of the downward shift and the upward trend indicates the bipolarization of flood impacts.
5. It detected the time lag between the peak of the numbers of flooded houses and the turning point in the ratio of the numbers to the inundated residential area.
6. Further, it showed that the annual flood damage in Japan was correlated with both the annual inundation area and the number of flooded houses. This suggested that reducing inundation water depth during a flood disaster would be effective in reducing flood damage. New countermeasures to reduce inundation depth will be a new challenge for Japan.
7. Rainwater storage is a good practice for reducing surface runoff and promoting public participation in flood risk management. Especially, the rain garden approach is a new initiative in Japan and is gaining momentum. However, the survey on cases in Kyoto indicated that the public involvement in this undertaking has been limited and the rain gardens have not penetrated into resident’s backyards.
8. A framework for promoting citizen science for flood risk management is proposed. It consists of three components: support from government finances, special technologies and mental support from workplaces; monitoring of flood impacts by residents using indices tangible to residents; promotion of measures taken by residents.
9. These results improved our understanding of flood impacts and enriched the literature by highlighting: (1) the right indicator should be chosen to detect significant changes; (2) the indicator identified in this study is tangible to residents, so it can play an important role in supporting and facilitating flood risk communication.

## Limitations and recommendation for further study

The present work is an exploratory study aimed at assessing changes in flood impacts from a perspective that is most tangible to residents. Once realized, it can be used to encourage public participation. Data analysis showed that properly normalized house flooding can meet such expectations. However, the applicability of such a perspective to other countries needs to be investigated. Furthermore, the drivers behind the identified change points should be further investigated. Besides, A shortcoming of the present study is that the number of flood disaster per year was not directly included in the analysis. In fact, the number of flooding per year was indirectly, although partially, considered in the present study because the annual inundation area is proportional to the annual number of flooding. Therefore, the ratio of the number of flooded houses divided by the annual inundated residential area accounts for the effect of flooding frequency to some extent. Since it is rare for a region to be affected by several major flood disasters in one year, including the annual number of flood disasters in the discussion and assessment can facilitate interregional dialogue across a nation, leading to better localized public participation in mitigating flood impacts.

Based on the results of the present study, the following recommendations are made for further studies.

1. Conduct a broad social survey to test the hypothesis that the use of house flooding is a better trigger than using annual national flood damage to engage the public into flood risk management.
2. Use the ratio of the number of houses flooded or semi-damaged divided by the flooded residential area to study the change in flood damage in other countries. In other words, generalization should be sought. One obstacle is data availability in many developing countries. To overcome this barrier, international cooperation will be a key. Developed nations can help developing countries establish reliable data-sharing systems, and can also use remote sensing technologies to help developing countries assess flood impacts
3. To study the mechanisms for the time lag between the peak of the number of flooded houses and the turning point in the ratio of the numbers to the inundated residential area.
4. To demonstrate that the framework for promoting public participation is working.
5. To explore ways for better flood disaster education to correct wrong perceptions such as the annual flooded area in Japan has been declining.
6. In addition to the use of annual impact data, the number of floods in each year should also be used in the analysis for better risk communication.
